# Ten-Year Changes in the Prevalence and Socio-Demographic Determinants of Physical Activity among Polish Adults Aged 20 to 74 Years. Results of the National Multicenter Health Surveys WOBASZ (2003-2005) and WOBASZ II (2013-2014)

**DOI:** 10.1371/journal.pone.0156766

**Published:** 2016-06-07

**Authors:** Magdalena Kwaśniewska, Małgorzata Pikala, Wojciech Bielecki, Elżbieta Dziankowska-Zaborszczyk, Ewa Rębowska, Krystyna Kozakiewicz, Andrzej Pająk, Jerzy Piwoński, Andrzej Tykarski, Tomasz Zdrojewski, Wojciech Drygas

**Affiliations:** 1 Department of Preventive Medicine, Medical University of Lodz, Lodz, Poland; 2 Department of Epidemiology and Biostatistics, Medical University of Lodz, Lodz, Poland; 3 Department of Social Pathologies, Medical University of Lodz, Lodz, Poland; 4 Department and Clinic of Cardiology, Medical University of Silesia, Katowice, Poland; 5 Department of Epidemiology and Population Studies, Institute of Public Health, Jagiellonian University of Krakow, Krakow, Poland; 6 Department of Epidemiology, Cardiovascular Disease Prevention and Health Promotion, Cardinal Stefan Wyszynski University, Institute of Cardiology, Warsaw, Poland; 7 Department and Clinic of Hipertensiology Angiology and Internal Medicine, Poznan University of Medical Sciences, Poznan, Poland; 8 Department and Clinic of Hypertension and Diabetology, Medical University of Gdansk, Gdansk, Poland; University of Bologna, ITALY

## Abstract

**Introduction:**

The aim of the study was to estimate ten-year changes in physical activity (PA) patterns and sociodemographic determinants among adult residents of Poland.

**Methods:**

The study comprised two independent samples of randomly selected adults aged 20–74 years participating in the National Multicentre Health Survey WOBASZ (2003–2005; n = 14572) and WOBASZ II (2013–2014; n = 5694). In both surveys the measurements were performed by six academic centers in all 16 voivodships of Poland (108 measurement points in each survey). Sociodemographic data were collected by an interviewer-administered questionnaire in both surveys. Physical activity was assessed in three domains: leisure-time, occupational and commuting physical activity.

**Results:**

Leisure-time PA changed substantially between the surveys (p<0.001). The prevalence of subjects being active on most days of week fell in both genders in the years 2003–2014 (37.4% vs 27.3% in men); 32.7% vs 28.3% in women. None or occasional activity increased from 49.6% to 56.8% in men, while remained stable in women (55.2% vs 54.9%). In both WOBASZ surveys the likelihood of physical inactivity was higher in less educated individuals, smokers and those living in large agglomerations (p<0.01). No significant changes were observed in occupational activity in men between the surveys, while in women percentage of sedentary work increased from 43.4% to % 49.4% (p<0.01). Commuting PA decreased significantly in both genders (p<0.001). About 79.3% of men and 71.3% of women reported no active commuting in the WOBASZ II survey.

**Conclusions:**

The observed unfavourable changes in PA emphasize the need for novel intervention concepts in order to reverse this direction. Further detailed monitoring of PA patterns in Poland is of particular importance.

## Introduction

Physical inactivity has been identified as the fourth leading risk factors for noncommunicable diseases and global mortality [1]. According to the latest report of Arem et al. (2015) physically inactive individuals have about 20% higher mortality risk as compared to those who meet current recommendations for physical activity (PA) [2]. Low PA has been also shown as an important determinant of metabolic disorders, cardiovascular diseases, cancers, depression and quality of life [2–7]. Despite the well known benefits of active lifestyle, globally about one third of a population aged 15 or older is physically inactive (i.e. not meeting current public health guidelines) [8].

Poland, a former communist country, is a unique example of dynamic political and socioeconomic transformation observed since 1988 followed by sudden, one of the fastest in the history, decline in mortality rates [9]. Several aspects of this phenomenon have been previously analyzed including contribution of lifestyle, risk factors as well as medical and surgical treatments. According to the report of Bandosz et al. (2012) over a half of the fall in mortality from coronary heart disease in Poland could be attributed to changes in major risk factors, mainly reduction in total cholesterol concentration and an increase in leisure-time physical activity [10]. Increased PA level explained about 10% of the decrease in mortality rates in Poland and resulted in about 2500 fewer coronary deaths between 1991 and 2005 [10]. Therefore, efforts focused on increasing PA level in Poland appear to be crucial in effective non-communicable diseases prevention and control. Monitoring temporal changes in PA patterns provides necessary information for elaborating appropriate health promotion strategies in target populations. Data for time trends in physical activity from low and middle-income countries are scarce. The National Multicentre Health Survey WOBASZ has been the largest and the most comprehensive Polish project allowing evaluation of several aspects of non-communicable diseases using standardized methods (validated questionnaires, standardized methods of risk factors measurement based on the Manual of Operations handbook of the WHO MONICA Project). The results of the WOBASZ survey carried out in 2003–2005 revealed an increase of PA level in Poland as compared with results obtained in the late 1990s [11]. The prevalence of sedentary lifestyle (i.e. none leisure-time PA declared) reached almost 80% among middle-aged Polish participants of the “Bridging the East-West Health Gap” Project [11]. The WOBASZ survey (2003–2005) revealed an improvement in PA level, as sedentary lifestyle was declared by 30% of the respondents [12,13].

The purpose of this report was to assess ten-year changes in PA patterns in adult residents of Poland. The present paper is the first report demonstrating changes in PA level in a representative national sample of Polish adults. Besides leisure-time PA, changes in occupational (work-related) and commuting (transportation) activity were also determined. Special attention was paid to sociodemographic determinants at the individual level and their potential association with PA level.

## Methods

The Ethical Committee of the Institute of Cardiology in Warsaw approved the study protocol. Informed written consent was obtained from each participant. Data were made anonymous before analysis.

### Study protocol and sample selection

For the purpose of this report we analyzed datasets taken from two nationwide representative cross-sectional surveys: WOBASZ carried out in the years 2003–2005 and WOBASZ II carried out in 2013–2014. The methodology of the surveys has been kept as similar as possible in order to enable a relevant monitoring of trends in collected data. A detailed description of the WOBASZ survey has already been published in previous papers [12–14].

For both surveys, an independent random sample was drawn from the national population register (adults aged 20–74 years in WOBASZ and adults aged ≥ 20 years in WOBASZ II). However, due to organizational and financial constraints, the random sample size of the WOBASZ II survey was smaller as compared with the previous survey (15 120 vs 19 200 individuals).

The two stages sampling scheme covered the whole territory of Poland and was stratified according to province and commune type. In each province of Poland 6 areas were selected (two rural, two small urban and two large urban areas) and next, in each area, samples of 70 men and 70 women aged 20 years and above years were selected using personal identification number. Personal invitations to participation in the study were sent to all chosen individuals. A total of 13.563 personal invitations were sent by post in the WOBASZ II survey.

Both WOBASZ surveys enrolled a total of 20 939 adults (14 769 participants in the WOBASZ and 6 170 participants in the WOBASZ II). The response rates were about 76% (WOBASZ) and 45.5% (WOBASZ II). Due to a relatively low response rate in the latest survey, we assessed the similarity index according to the distribution of age and educational level using current nationwide sociodemographic data for Polish adults [15] and the data gathered within the WOBASZ II survey. According to the Central Statistical Office, the proportion of young adults (18–34 years) and persons with elementary education decreased while the proportions of seniors (above 64 years) and persons with university education increased in Poland in the years 2002–2013 [16]. The same trend was found in the distribution of these variables among the participants of our surveys. The similarity index was 91.2% for age and 88.6% for educational level between the Central Statistical Office data and the WOBASZ II sample.

As the age range of the participants in the first edition of WOBASZ Study was 20–74 years, the subjects aged above 74 years old were excluded from WOBASZ II database for the purpose of this analysis. After excluding subjects with incomplete data on the required questions, the final sample comprised 8545 men (5943 in WOBASZ and 2602 in WOBASZ II) and 9755 women (6609 in WOBASZ and 3146 in WOBASZ II) aged 20–74 years and above. All procedures were carried out by nurses and trained interviewers in the participants’ houses or in selected out-patient clinics and were comparable in both WOBASZ surveys. The methodology has closely followed the WHO MONICA protocol [17], and consisted of the following parts: a questionnaire interview, blood pressure, heart rate and anthropometric measurements, and a blood sample collection. The WOBASZ questionnaire included detailed questions on medical history, sociodemographic and economic factors, health knowledge, attitudes, lifestyle, nutrition, social support and depression. All employed interviewers were trained in the application, completion and codification of the questionnaire. Fieldwork supervisors conducted controls in the selected samples of interviewers.

In the present analysis the following sociodemographic measures were taken into account: age, residential status, educational level, marital status, smoking. The participants were divided into the three categories of place of residence according to the number of inhabitants in their living area (“rural” area < 8 000 inhabitants; small urban area 8 000–40 000 inhabitants; large urban area >40 000 inhabitants). Educational level was categorized as elementary (no education/primary school), secondary (high school vocational/ incomplete high school/high school/vocational higher than high school) and university attainment (incomplete university/complete university education). All the analysed socio-demographic factors were thought as factors related to PA level of and therefore called “contributing factors”.

### Assessment of leisure time, occupational and commuting physical activity

Physical activity assessment was based on the WHO MONICA protocol and CINDI Health Monitor Questionnaire [17]. Similar set of questions was used in previous studies carried out in Polish population [11,13,18]. Self-reported data on PA were assessed in three domains: leisure-time, commuting (transportation) and occupational (work-related) physical activity.

Physical activity in leisure time was defined as regularly doing physical exercises accumulating at least 30 minutes per day. The participants were asked: “Do you regularly do physical exercises (for ex. running, walking, swimming, cycling, gymnastics, gardening etc.) for at least 30 minutes per day?” The possible answers were: “yes” or”no”. Those who answered “yes” were asked: “How often are you physically active?” There were six possible answers: “everyday”, “4–6 days per week”, “Every second or every third day per week”, “once a week”, “two or three times per month”, “once a month or less frequent”. Individuals who did not declare doing any physical exercises in their leisure time were defined as “physically inactive” and asked about the reasons of sedentary lifestyle.

Occupationally active respondents were asked: “What is the kind of your work?”. There were three possible answers: “mainly sitting or standing work (more than a half of time spent on sitting/standing), “mainly heavy physical work (more than a half of time spent on heavy physical work)” “other, not defined by the above answers”.

Therefore occupational PA was assessed according to the following three categories: “low” (mostly sitting or standing office work), “moderate” (light/moderate physical work) and “high” (heavy manual work).

Commuting PA was measured by asking the participants whether they walked, rode a bicycle, or used motorized transportation to/from work. The daily commuting return journey was categorized into four possibilities: using motorised transportation; walking/bicycling 1–14 min; 15–29 min or ≥ 30 min. The following questions were used in the questionnaire: “How do you usually travel to and from your work/school? There were three possible answers: “By means of public transport”, “by car”, “walking or bicycling”. Those who declare active commuting were asked: “How much tim How much time do you spent walking or cycling to and from your work/school per day?” There were four answers: “less than 15 minutes “, 15–30 minutes”, “31–60 minutes”, “more than 60 minutes”.

### Statistical analysis

To compare the frequency and assess statistical significance of the categories of qualitative characteristics in the analysed groups the chi-square test was implemented. Additionally to p-values, omega-squared formula (ω^2^) was implemented as an effect size. Given that the potential correlates might differ between genders, all the analyses were performed separately for men and women. In order to eliminate the potential influence of age in the calculations, a direct standardization was implemented, following the Polish population structure as of 31.12.2013 [14]. In order to identify socio-demographic factors (age, place of residence, educational level, marital level) that can contribute to physical inactivity, logistic regression analysis was performed. The results were shown as odds ratios (OR) with 95% confidence intervals (CI) for being inactive in leisure time. Individuals aged <35 years, residents of rural settings, with university education, single and not smoking were used as a reference, being assigned an OR value of 1.00. The multivariate logistic regression analyses were adjusted for age, education, place of residence, smoking and other domains of PA. All p values were two-sided and p<0.05 was set as statistically significant. Statistical analyses were performed using STATISTICA Windows XP version 12. Data used in this analysis are available at S1 File.

## Results

**Fig 1:**
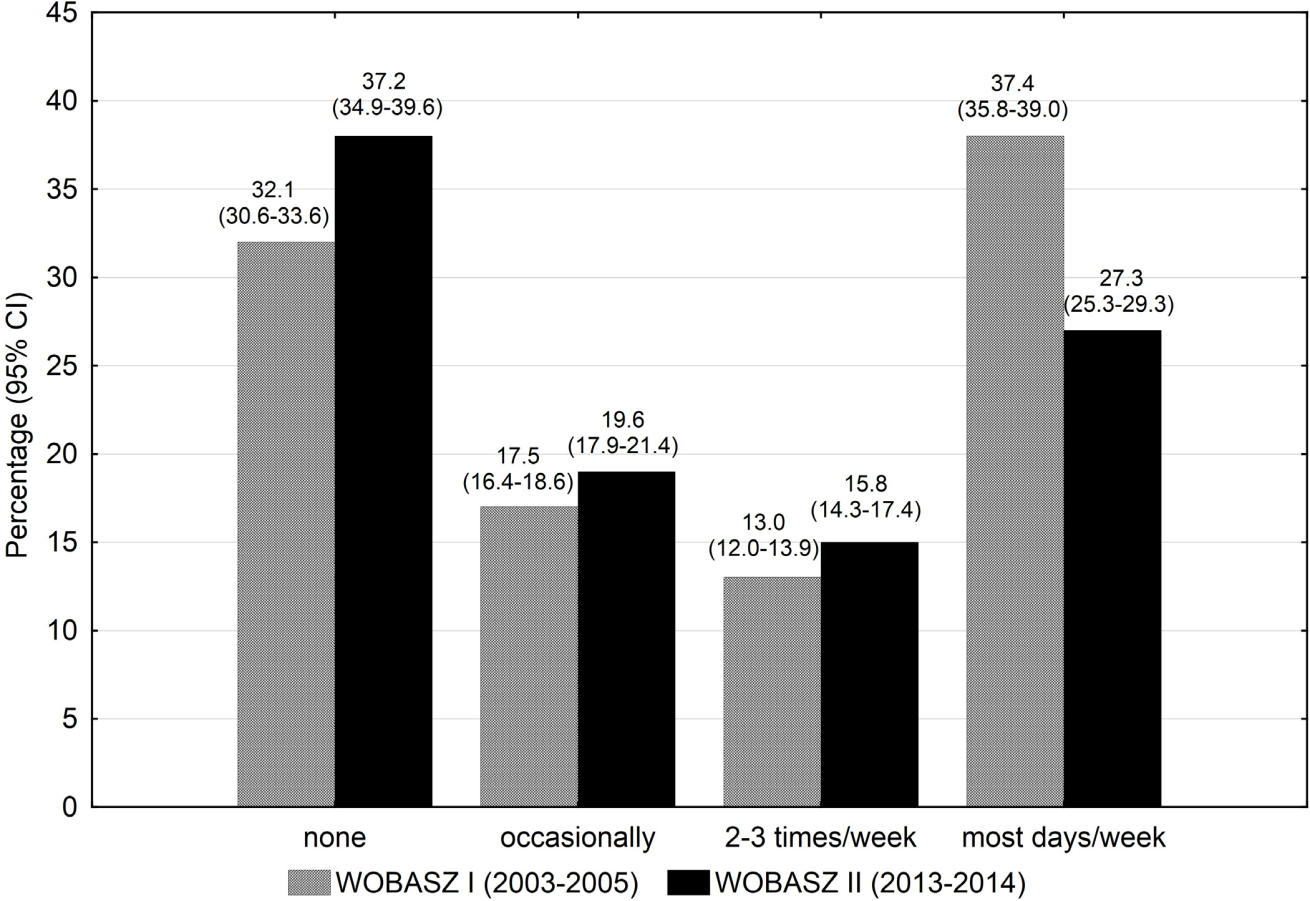
Ten-year changes in leisure time physical activity (exercises lasting > = 30 min/day) among men participating in the WOBASZ surveys.

**Fig 2:**
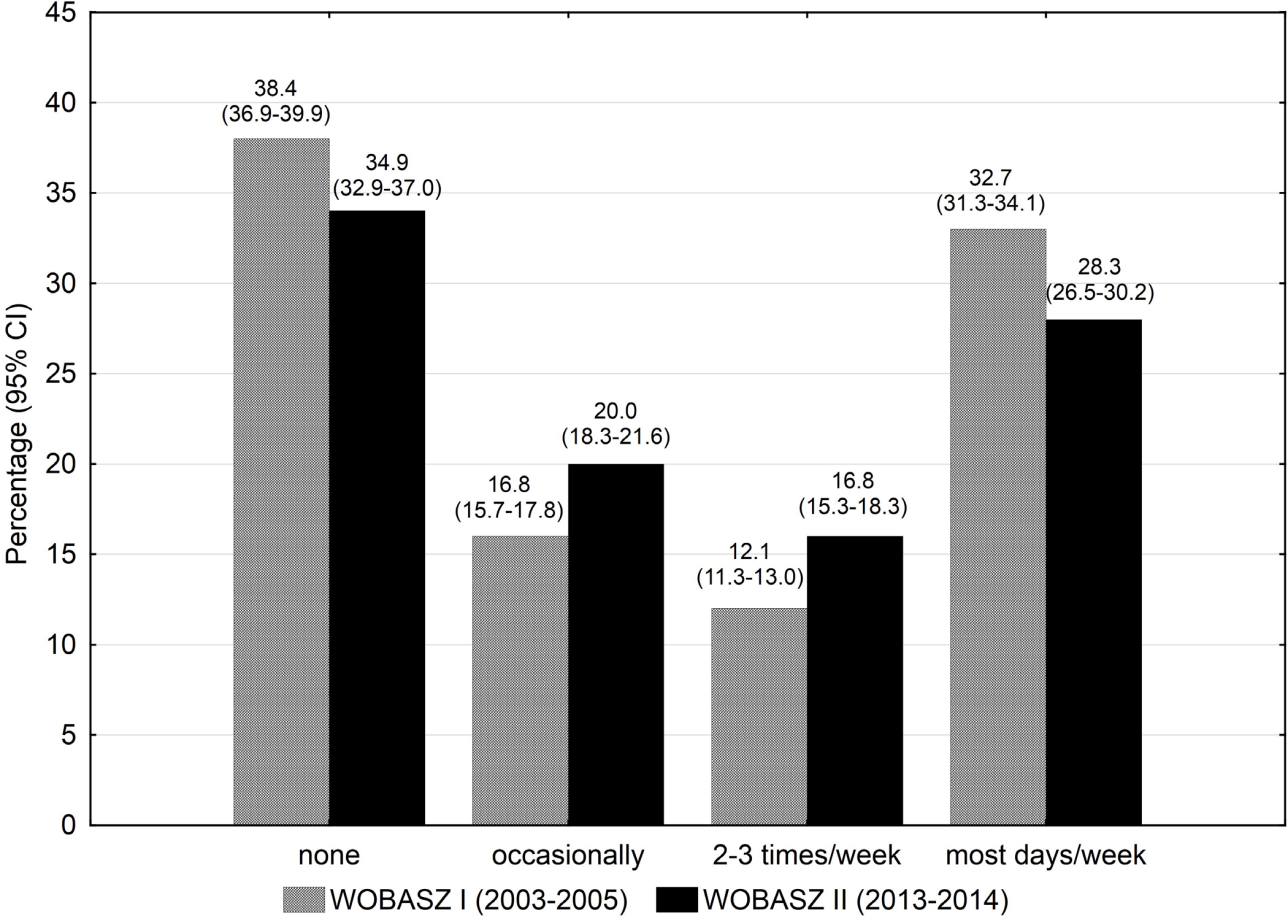
Ten-year changes in leisure time physical activity (exercises lasting > = 30 min/day) among women participating in the WOBASZ surveys.

The general sociodemographic characteristics of the studied populations are presented in Table 1. Leisure-time PA level changed substantially between the surveys in both genders (p<0.001, ω^2^ = 0.1)(Figs 1 and 2). The prevalence of subjects being active on most days of week decreased in both genders between the surveys (Figs 1 and 2). None or occasional PA increased from 49.6% to 56.8% in men, while remained fairly stable in women (55.2% vs 54.9%) in the analysed period of time.

**Table 1:** Sociodemographic characteristics of the studied population in the WOBASZ (2003–2005) and WOBASZ II (2013–2014) Survey.

	Men n = 8545				Womenn = 9755			
	WOBASZ n = 5943		WOBASZ II n = 2602		WOBASZ n = 6609		WOBASZ II n = 3146	
	n	(%)	n	(%)	n	(%)	n	(%)
**Age (years)**								
20–34	1390	(23.4)	653	(25.1)	1574	(23.8)	723	(23.6)
35–64	3643	(61.3)	1597	(61.4)	4078	(61.7)	1992	(62.2)
>64	910	(15.3)	352	(13.5)	957	(14.5)	431	(14.2)
**Place of residence**								
Rural	1980	(33.3)	851	(32.7)	2218	(33.6)	1089	(34.6)
Small urban	1925	(32.4)	781	(30.0)	2158	(32.6)	958	(30.5)
Large urban	2038	(34.3)	970	(37.3)	2233	(33.8)	1099	(34.9)
**Educational level**								
Elementary	1357	(22.8)	300	(11.5)	1737	(26.3)	443	(14.1)
Secondary	3834	(64.5)	1728	(66.4)	3702	(56.0)	1588	(50.5)
University	752	(12.7)	574	(22.1)	1170	(17.7)	1115	(35.4)
**Marital status**								
Single	1010	(17.0)	598	(23.0)	676	(10.2)	450	(11.5)
Married	4606	(77.5)	1810	(69.6)	4805	(72.7)	2126	(71.1)
Divorced	176	(3.0)	127	(4.9)	313	(4.8)	225	(5.5)
Widowed	151	(2.5)	67	(2.5)	815	(12.3)	345	(11.9)

Analysis of distribution of leisure-time PA according to age revealed that the percentage of physically active on most days of week fell in young and middle-aged adults between the surveys (30.9% vs 26% and 36.5% vs 28% in the groups aged 18–34 and 35–64 years old, respectively). Among seniors aged 65–74 years the proportion of the most physically active individuals remained stable (36.7 vs 36%)(data not shown in the tables).

The most common factors of sedentary lifestyle declared by the participants were lack of time (26.% and 30.0% in the subsequent surveys, respectively) and no interest in doing exercises (24% and 21.1%). In the latest survey significantly more persons reported that high occupational activity was an important factor of physical inactivity (14.8% vs 17.7% in men, p<0.001 and 6.2% vs 8.0%, p<0.001 in women in 2003–2005 and 2013–2014, respectively).

**Fig 3:**
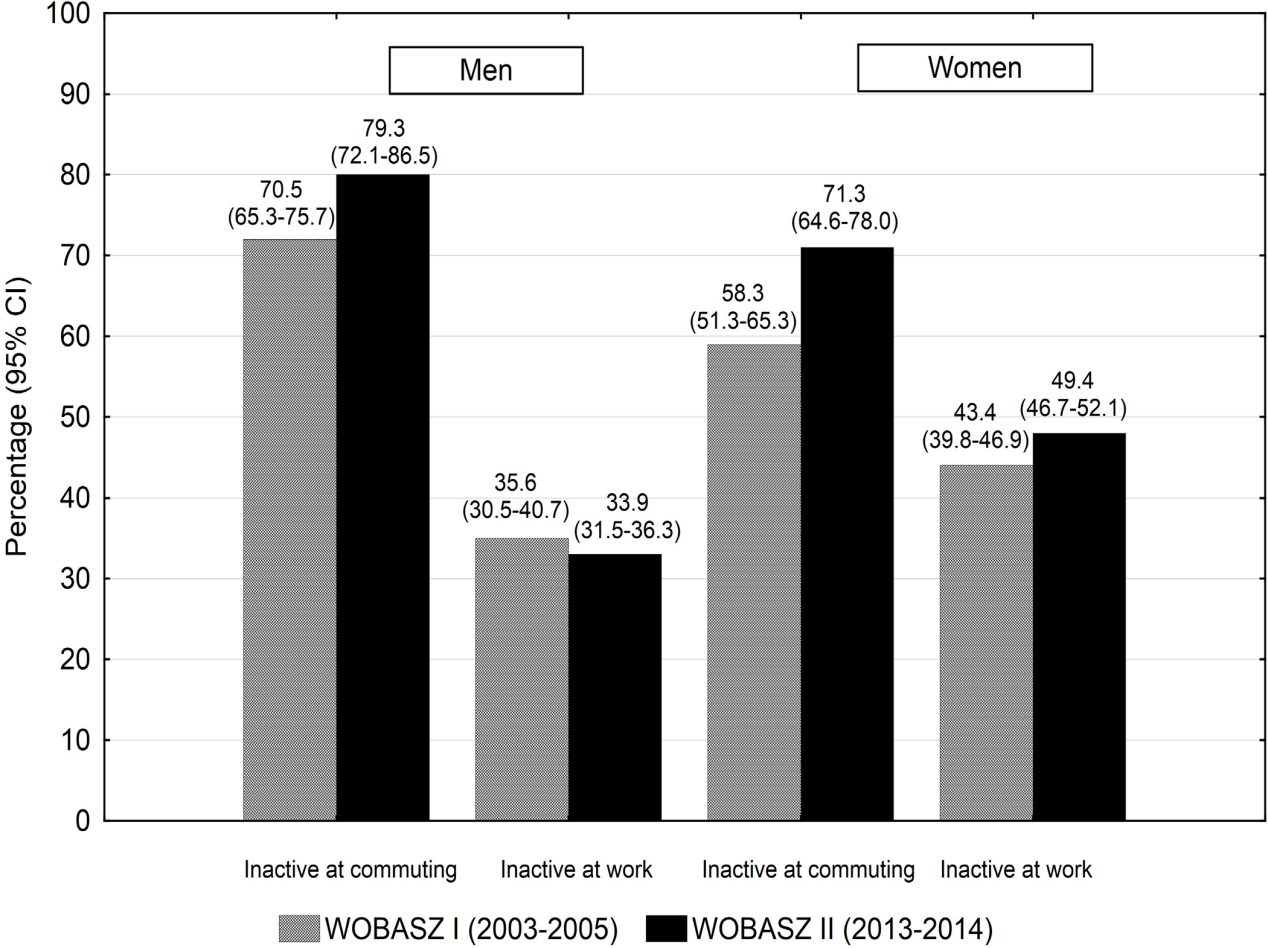
Changes in commuting and occupational inactivity among the participants of the WOBASZ surveys.

The analysis of occupational PA revealed significant differences in a distribution of work-related PA among women (p<0.001, ω^2^ = 0.05). Prevalence of women declaring sedentary work increased from 43.4% to 49.4% (Fig 3). High occupational PA was declared by 45.8% of men and 26.2% of women and by 44.8% of men and 28.4% of women in the WOBASZ and WOBASZ II surveys, respectively (p<0.001 for a comparison between genders). Commuting PA changed substantially in the years 2003–2014. Prevalence of persons usually using motorised transportation increased from 70.5% to 79.3% in men (p<0.001) and from 58.3% to 71.3% in women (p<0.001) (Fig 3).

As shown in Table 2, physical inactivity in leisure time was significantly related to various sociodemographic and behavioural characteristics. Age occurred a significant predictor of physical inactivity among men in both surveys. Young men had generally lower probability of inactivity as compared to older participants of the study. However, in the latest survey the highest prevalence of physical inactivity was found among middle-aged persons (Table 2). Physical inactivity was above 2-fold higher in persons with elementary education as compared to individuals with university attainment (p<0.01). Regular smokers were less physically active as non-smokers in both genders. High occupational PA remained significantly associated with a higher prevalence of inactivity in leisure time among men during the observation (p<0.01).

**Table 2:** Odds Ratios (OR)^a^ and 95% Confidence Intervals (CI) for inactivity during leisure-time to sociodemographic and PA characteristics among participants of the WOBASZ (2003–2005) and WOBASZ II (2013–2014) surveys.

Characteristics	Men				Women			
	WOBASZ		WOBASZ II		WOBASZ		WOBASZ II	
	OR	95%CI	OR	95%CI	OR	95%CI	OR	95%CI
**Age (years)**								
<35 (referent)	1.00		1.00		1.00		1.00	
35–64	1.18	1.10–1.41*	1.47	1.17–1.86**	0.90	0.75–1.01	1.05	0.85–1.30
>64	1.35	1.14–1.73**	1.10	0.80–1.53	1.35	1.14–1.73**	1.25	0.93–1.69
**Educational level**								
Elementary	1.64	1.31–2.11**	3.64	2.62–5.06**	1.65	1.11–2.12**	2.52	1.94–3.27**
Secondary	1.31	1.22–1.64**	2.00	1.59–2.50**	1.32	1.20–1.54**	1.63	1.37–1.94**
University (referent)	1.00		1.00		1.00		1.00	
**Place of residence**								
Rural (referent)	1.00		1.00		1.00		1.00	
Small urban	0.91	0.57–0.98*	0.75	0.61–0.92**	0.91	0.57–0.98*	0.99	0.82–1.19
Large urban	0.87	0.54–0.89*	0.72	0.59–0.88**	1.47	1.24–1.61**	1.33	1.10–1.59**
**Marital status**								
Single (referent)								
Married	0.95	0.60–1.0	0.91	0.73–1.15	1.75	1.16–2.1**	1.48	1.15–1.90**
Divorced	0.90	0.57–1.17	0.69	0.45–1.06	0.79	0.57–1.17	1.59	1.10–2.30**
Widowed	1.0	0.54–1.3	0.83	0.48–1.46	0.73	0.54–1.30	1.80	1.28–2.54*
**Smoking**								
no (referent)	1.00		1.00		1.00		1.00	
yes	1.26	1.13–1.41**	1.37	1.14–1.64**	1.17	1.04–1.32**	1.42	1.18–1.70**
**Occupational physical activity**								
Low (referent)	1.00		1.00		1.00		1.00	
Moderate	1.39	1.15–1.69**	1.20	0.94–1.53	1.53	1.11–1.79**	0.95	0.77–1.17
High	1.41	1.19–1.75**	1.68	1.36–2.09**	1.58	1.12–1.87**	1.09	0.89–1.33
**Commuting physical activity (min/day)**								
0 (referent)	1.00		1.00		1.00		1.00	
1–14	1.05	0.57–1.32	0.85	0.60–1.19	1.10	0.27–1.29	0.92	0.69–1.23
≥15	1.02	0.43–1.29	0.92	0.60–1.42	1.17	0.38–1.31	1.08	0.77–1.52

* p < 0.05

** p<0.01

^a^ Adjusted OR took account of age, place of residence, education, marital status and other forms of physical activity.

Additional analysis was performed in order to assess the interrelations of the three types of PA. The percentage of persons highly active in all the analysed domains (i.e. leisure-time PA on most days of week, high occupational PA and active commuting 15 min or more per day) decreased in both genders in the studied period (men: 3.1% and 0.5%; women: 1.8% and 0.9% in 2003/5 and 2013/14, respectively). On the other hand, the prevalence of the least active individuals (no leisure-time, low occupational PA and no active commuting PA) did not change substantially between the surveys (men: 9.0% and 9.4%; women: 13.9% and 12.3% in 2003/5 and 2013/14, respectively). In the latest survey, among all individuals active at work about 7.2% of men and 15.7% of women were also active in leisure-time on most days of week.

## Discussion

The present report demonstrates ten-year changes in physical activity patterns among adults in Poland. The obtained results suggest that remarkable favorable trends, which Poland has experienced over the past decades, seem to weaken in the recent years.

Although the proportion of inactive women remained fairly stable over time, a significant increase of physical inactivity in leisure-time was observed among men. Moreover, the percentages of subjects physically active on most days of week fell substantially between the surveys in both genders. Unfavorable changes occurred also in commuting activity.

Majority of the studies investigating time trends in leisure-time PA have come from high-income countries. Adults participation in regular exercises tend to be increasing over time in several countries, including Canada, England, Spain or Finland [19–23]. A spectacular increase in PA was observed in Finland where high level of leisure-time PA increased from 21% to 33% in men and from 12% to 27% in women between 1982 and 2012 [23]. Among countries of Central and Eastern Europe, the Czech Republic seems a relatively active society as more than 46% of adults declare sufficient physical activity [24,25].

Previous surveys including Polish adults provided inconsistent results [18, 26–29]. The European Health Interview Survey showed that every fourth Pole aged 15 and older is physically active on everyday basis [26]. However, according to a subsequent comparative analysis including 15 European Union Member States, the prevalence of adults involved in everyday exercises increased to 53.4% [27]. Meanwhile, the findings of the Nationwide Study of Occurrence of Risk Factors of Cardiovascular Diseases (2011) demonstrated that above 39% of adults was physically active on most days of week [18]. The latest data on PA level in the European Union place Polish adults much below the European average. About 28% of Poles declared practicing vigorously sport at least once a week, while for example in Finland or Denmark, the proportions reach 66% and 68%, respectively [28]. The observed heterogeneity of the data concerning PA level in Poland probably result from discrepancies in methodology, including the number of participants, sociodemographic characteristics, local approach or different techniques and definitions of measuring PA.

Prevalence of physical inactivity in leisure-time increased from about 35% to more than 38% during ten-year observation in our country [29]. Data concerning physical inactivity obtained in other countries of our region are also unsatisfactory. Brazil seems quite a similar example of unfavorable trends in PA level as the prevalence of insufficiently active adults (not meeting current recommendations) increased from 41.1% to 54% between 2002 and 2012 [30]. The report of Hamrik et al. (2014) showed that about 32.3% of adult Czechs declared a low level of PA, while more than 60% across the age categories were rated as “sedentary” [24]. Importantly, a remarkable decrease in the prevalence of adequate physical activity level Czech adults has been observed in recent years as compared with previous studies [31]. Another European survey demonstrated that about 32% of Hungarians and 73.2% of Romanians are insufficiently active [32].

Generally, PA level is strongly related to several sociodemographic variables. It has been documented that lower PA is more prevalent in older individuals and with lower socioeconomic status [8,33]. However, the most striking results of the WOBASZ II survey concern the highest prevalence of physical inactivity among middle-aged men. These findings are of particular importance due to still high premature mortality among men in our country. According to the reports of Maniecka-Bryła et al., the greatest number of years of life lost in males at productive age were due to ischemic heart disease [34,35]. Therefore, it would be most beneficial to focus on decreasing major risk factors of CVD, including physical inactivity, in order to improve life expectancy in Poland. Consistently with several previous reports, the highest prevalence of low leisure-time PA was observed among persons with elementary education in both WOBASZ surveys [11,24,29].

Another important issue concerns promoting PA among older individuals. Although the percentage of seniors doing exercises on most days of week was higher than in the youngest age group, the proportion of physically active individuals aged 64–74 years remains still unsatisfactory. The observed results are consistent with the latest findings of the PolSenior Project which showed that about 30% of seniors met current recommendations for PA [36]. Rowinski et al. (2015) demonstrated that there were substantial differences in the leisure-time PA of older adults in Poland compared with those in North America or Western Europe [36]. Thus, public health messages and environmental policies should concentrate on raising awareness of the importance of PA and motivation for exercising for the elderly. Gardening, cycling and walking seem potentially the most reliable and attractive forms of leisure-time PA for Polish seniors [36].

Regular smokers are still significantly less physically active than non-smokers which additionally increases their risk of morbidity and mortality due to chronic non-communicable diseases. Physical activity, especially vigorous leisure-time PA might help prevent disability retirement not only among never-smokers, but even among ex-smokers and moderate smokers [37]. Among heavy smokers, any PA is sufficient to eliminate the adverse effects of smoking on health and thus, there is no doubt that decreasing prevalence of smoking remains among public health priorities.

A systematic decrease in occupational PA seems to be an overall trend, especially in middle and high-income societies [8]. Several researches also reported a reduction in occupational PA over time which is, to some extent, consistent with our findings [19,20,22,38]. In the United States, work-related energy expenditure has fallen by more than 100 calories per day in last 50 years [38]. As expected, work-related PA remained significantly higher among men as compared with women in both WOBASZ surveys. Importantly, we noted an increasing number of persons declaring that high occupational PA is an important contributor of low PA in leisure time. Indeed, according to Organisation for Economic Co-operation and Development (OECD) and Eurostat data, Polish adults are among the most hardworking societies (about 40.1 hours/week spent at work in 2011).

Active commuting is still not common in our country and contributes only slightly to total PA. About 9% of all active commuters in both surveys regularly walked/cycled for at least 30 min/day and, therefore, adhered to the current guidelines regardless of leisure-time PA. We can also suppose that those who declared leisure-time activities at least 30 minutes 2–3 times per week might achieve the recommended level of total PA by active transportation lasting at least 15 min/day. This could concern about 4% of all active commuters.

The observed increase in motorized transportation to and from work may be a result of socioeconomic changes and economic growth in Poland in recent years. On the other hand, policies promoting modern infrastructure and a safe environment for bicycling have substantially developed in Poland in the past decades. Each year a number of new facilities is largely available giving opportunity for commuting other than using cars and motorcycles. Since active commuting provide substantial health benefits [39–41], a decline of this kind of PA occurs particularly disturbing and need a special attention while elaborating future interventions. The proportions of active commuters in other countries range from below 5% in the United States, Australia or Switzerland to much above 20% in Denmark, Sweden or the Netherlands [8]. While analyzing data on transportation PA in Brazil, unfavorable trends have been observed since 2006 which is in line with our data [42].

A certain paradox in the context of lifestyle in our country should be pointed out. Although the general PA level has been shown to decrease, a number of persons participating in mass sports events is constantly increasing [43]. All organized marathon races or other running competitions on shorter distances (5 and 10 km) gather thousands of enthusiasts each year. The events are usually well supported by visible mass media. It seems, however, that this kind of activity does not correspond with total PA of general Polish society.

Despite a noticeable increase of interventions aimed at promoting active lifestyle, the long-term effectiveness of the implemented strategies in Poland remains unsatisfactory. Majority of programs adopted a local and short-term approach. A few large national projects, including The Great Nationwide Physical Activity Campaign "Revitalize Your Heart", although successful, had to be discontinued due to financial reasons [44]. Perhaps, elaboration of the stable and consistent national policy for PA promotion, including cooperation between private and public sector, might result in achieving favorable long-lasting trends. High prevalence of non-communicable diseases, unsatisfactory PA level and the lack of national strategy for PA promotion apply also to other countries in our region [45].

Some limitations of the study should be recognized. Self-reported measurement of PA may be associated with recall bias. Moreover, a precise quantification of PA level was not possible with the questionnaire used in our study. Using accelerometers and calculation exercise-related energy expenditure would certainly improve the study [46]. However, this method is rarely used in large-scale population studies, especially in low or middle-income countries. the questions on leisure-time PA considered only exercises lasting at least 30 minutes per day. We could not, therefore, assess how many persons were engaged in shorter exercise sessions. Another limitation concerns a decrease in response rates between the surveys. Declining participation rates are an increasing problem in most societies and demands searching for novel methods of motivating invitees to participate [47]. Perhaps a combinations of phone calls, invitation letter and home visits would be new effective methods to recruit invitees. Moreover, using incentives, websites and promotion in local media to promote the surveys might also be useful. There are several variables related to lifestyles and health profile that could be analyzed in this manuscript. However, as we are preparing another study concerning the lifestyle index (based on nutrition, physical activity, smoking, alcohol consumption and anthropometric data), in the present paper we focused on the selected sociodemographic variables.

Finally, the results obtained in this analysis may be relevant only for adults aged 20–74 years old.

There are important strengths of this study. This is the first analysis providing data on change in PA level over time in a large national representative sample of adults in Poland. Besides leisure-time PA, also occupational and commuting patterns were taken into account. Of note, both WOBASZ surveys adopted consistent methodology to ensure comparable data on trends in analyzed variables. Our findings could be of particular importance for other middle-income countries.

## Conclusions

Although it is too early to conclude whether this is the beginning of a constant disturbing trend or just a temporary decline of PA in Poland, the results of this analysis raise concerns and prompt to consider novel intervention concepts. More specific approach is required for the most vulnerable sociodemographic groups, i.e. middle-aged men, older persons (especially women) and less educated individuals. There are several potentially effective methods of increasing PA level at the population level including engagement of occupational health specialists, family practitioners, trained nurses and health educators. The observed unfavorable changes in PA level emphasize the importance of further detailed monitoring of PA patterns in Poland.

## Supporting Information

S1 File Dataset of the WOBASZ Project. (XLS)
